# Evolution of C-Reactive Protein

**DOI:** 10.3389/fimmu.2019.00943

**Published:** 2019-04-30

**Authors:** Asmita Pathak, Alok Agrawal

**Affiliations:** Department of Biomedical Sciences, James H. Quillen College of Medicine, East Tennessee State University, Johnson City, TN, United States

**Keywords:** C-reactive protein, pentraxin, serum amyloid P, phosphocholine, PTX3

## Abstract

C-reactive protein (CRP) is an evolutionarily conserved protein. From arthropods to humans, CRP has been found in every organism where the presence of CRP has been sought. Human CRP is a pentamer made up of five identical subunits which binds to phosphocholine (PCh) in a Ca^2+^-dependent manner. In various species, we define a protein as CRP if it has any two of the following three characteristics: First, it is a cyclic oligomer of almost identical subunits of molecular weight 20–30 kDa. Second, it binds to PCh in a Ca^2+^-dependent manner. Third, it exhibits immunological cross-reactivity with human CRP. In the arthropod horseshoe crab, CRP is a constitutively expressed protein, while in humans, CRP is an acute phase plasma protein and a component of the acute phase response. As the nature of CRP gene expression evolved from a constitutively expressed protein in arthropods to an acute phase protein in humans, the definition of CRP became distinctive. In humans, CRP can be distinguished from other homologous proteins such as serum amyloid P, but this is not the case for most other vertebrates and invertebrates. Literature indicates that the binding ability of CRP to PCh is less relevant than its binding to other ligands. Human CRP displays structure-based ligand-binding specificities, but it is not known if that is true for invertebrate CRP. During evolution, changes in the intrachain disulfide and interchain disulfide bonds and changes in the glycosylation status of CRP may be responsible for different structure-function relationships of CRP in various species. More studies of invertebrate CRP are needed to understand the reasons behind such evolution of CRP. Also, CRP evolved as a component of and along with the development of the immune system. It is important to understand the biology of ancient CRP molecules because the knowledge could be useful for immunodeficient individuals.

## Introduction

Human C-reactive protein (CRP) was identified as a plasma protein which, in the presence of Ca^2+^, precipitated C-polysaccharide (PnC) isolated from the cell wall of *Streptococcus pneumoniae* (1). The precipitation was due to the binding of CRP to phosphocholine (PCh) moiety present in PnC (2). In animals, we define a protein as CRP if it has at least two of the following three characteristics: First, it is a cyclic oligomer of almost identical subunits of molecular weight 20–30 kDa. Second, it binds to PCh in a Ca^2+^-dependent manner. Third, it exhibits immunological cross-reactivity with human CRP.

**Figure 1:**
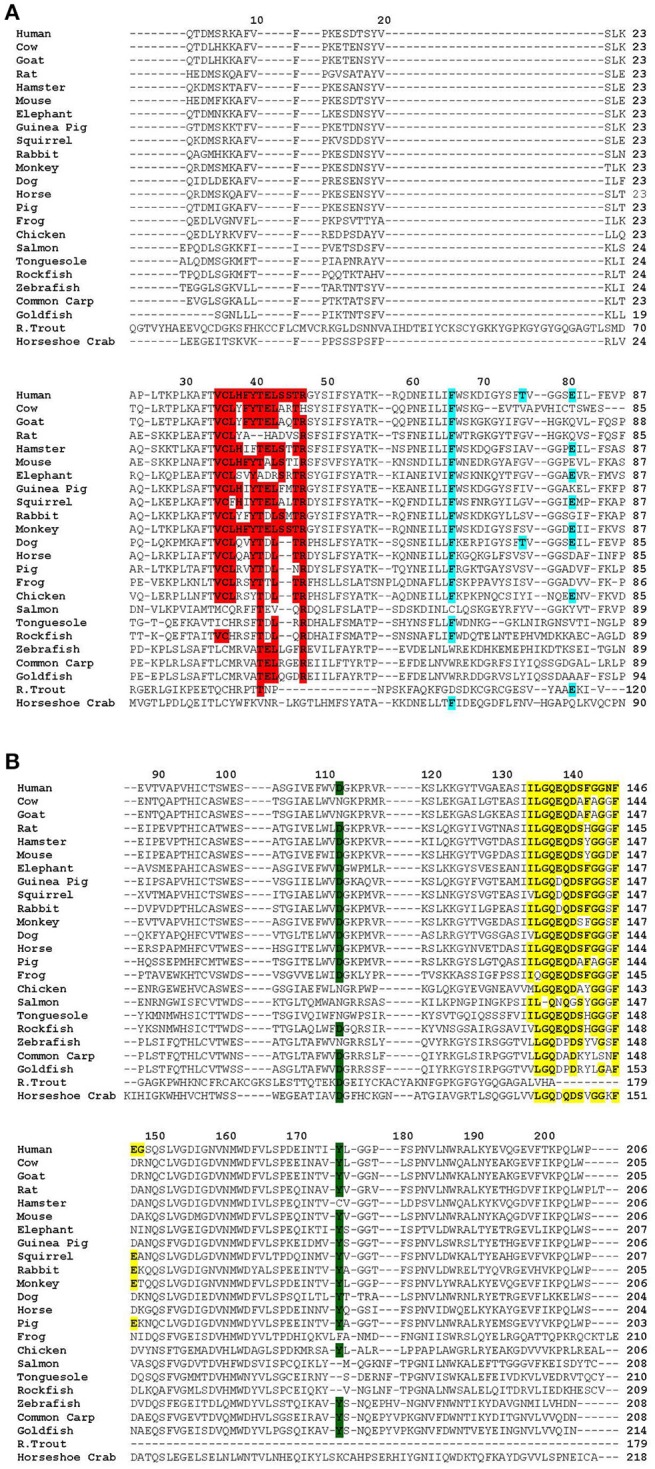
Alignment of the primary structure of CRP from various species using Clustal Omega (1.2.4) EMBL-EBI multiple sequence alignment software. Sequences were obtained from NCBI in FASTA format and copied into the EMBL-EBI alignment software where the output result was obtained in the format of ClustalW with character counts. For horse and horseshoe crab, there were several sequences due to the presence of isoforms. Since the microheterogeneity between these isoforms was <10%, the first isoform sequence was selected. Accession numbers of the sequences are: Horseshoe crab, AAA28270; Rainbow trout, NP001118197.1; Goldfish, AK022072.1; Common carp, AEU04519; Zebrafish, AGB69036; Rockfish, AKR17056; Tonguefish, NP001281151; Salmon, NP001134140; Chicken, ABD16281; Frog, NP001165686; Pig, NP999009; Horse, XP001504452; Dog, CDF47287; Monkey, XP001117250; Rabbit, NP001075734; Squirrel, XP026263752.1; Guinea pig, AAC60662; Elephant, XP006895510.1; Mouse, AFA37877; Hamster, XP005078251; Rat, AFA37869; Goat, XP017901842; Cow, NP001137569; and Human, AAL48218. The sequence of the signal peptide is not shown. The column on the right shows the number of amino acid residues in each CRP. **(A)** Sequence of amino acid residues 1–87 of human CRP aligned with the sequence of CRP from other animals. Conserved amino acid residues in the following functional sites are highlighted: The intrinsically disordered region in CRP (amino acid residues 35–46 in human CRP) is highlighted in red. The PCh-binding site (amino acid residues 66, 76, and 81 in human CRP) is highlighted in blue. **(B)** Sequence of amino acid residues 88–206 of human CRP aligned with the sequence of CRP from other animals. Conserved amino acid residues in the following functional sites are highlighted: The C1q-binding site (amino acid residues 112 and 175 in human CRP) is highlighted in green. The Ca^2+^-binding site (amino acid residues 134–148 in human CRP) is highlighted in yellow.

CRP is an evolutionarily conserved protein. From arthropods to humans, CRP has been found in every organism where the presence of CRP has been sought (3–8). In the arthropod horseshoe crab, CRP is a constitutively expressed protein found in the hemolymph (8). After ~500 million years of evolution, in humans and some other species, CRP became a protein which is expressed as a component of the acute phase response (9). The aim of this paper was to review the changes observed in the structure and ligand-binding specificities of CRP during evolution. We reviewed the literature on the structure and ligand-binding specificities of CRP from the following animals from arthropods to humans: American horseshoe crab, giant African snail, 17 different species of fish, chicken, frog, cow, dog, guinea pig, horse, hamster, mouse, goat, rat, rabbit, monkey, pig, mink, elephant, squirrel, seal, phascogale, and man. We compared the primary structure of CRP and searched for the conservation of functionally critical amino acid residues that are known for human CRP (Figure 1). We also compared the overall quaternary structure (Table 1), ligand-binding specificities, and immunological cross-reactivity of CRP (Table 2), if known. Two other proteins, serum amyloid P component (SAP), also known as pentraxin-2, and long pentraxin (PTX3), which share several structural and functional properties with CRP, are not reviewed here (3, 56).

**Table 1:** Properties of CRP isolated from various animals. See the text for scientific names of the animals.

**Animal**	**Approximate molecular weight (kDa)**	**Symmetry (quaternary structure)**	**Approximate molecular of subunits (kDa)**	**Glycosylation**	**Acute phase protein**	**References**
Horseshoe crab	300	Hexamer (stack of two hexamers)	25	Yes	No	(10–13)
Giant African Snail	400		20 and 24	Yes	No	(14, 15)
Plaice	187	Pentamer (10 subunits)	18.7	Yes	No	(16–19)
Lumpsucker	125–150	Pentamer	20–21.5			(20, 21)
Rainbow trout	81.4	Trimer	26.6	Yes	Yes	(22)
Cod		Pentamer	22–29	Yes		(23, 24)
Eel	120	Pentamer	24	No		(25, 26)
Channel catfish	100	Pentamer		No	Yes	(27)
Striped catfish		Trimer	28	Yes		(28)
Ayu			25.2	No	Yes	(29)
Rohu		Pentamer	33	Yes	Yes	(30)
Common carp			27	Yes	Yes	(31)
Major carp			22 and 29	Yes	Yes	(32, 33)
Goldfish			25.6		Yes	(34)
Dogfish	250	Pentamer of dimers	25			(35, 36)
Zebrafish		Trimer			Yes	(37, 38)
Tonguefish			26		Yes	(39)
Rockfish	160 and 152		30 and 26	Yes	Yes	(40)
Frog			24		No	(41)
Cow	100	Pentamer	23	No	No	(42, 43)
Dog	115	Pentamer	21	Yes	Yes	(44, 45)
Harbor seal			25		Yes	(46)
Goat	120	Pentamer	24	Yes	No	(47)
Horse	118	Pentamer	24	No	Yes	(48)
Hamster	128–150	Pentamer	27–30	Yes	Yes	(49–51)
Rabbit	115–140	Pentamer	23.5	No	Yes	(52, 53)
Rat	129	Pentamer	23	Yes	Yes	(54)
Human	115	Pentamer	23	No	Yes	(55)

**Table 2:** Immunological cross-reactivity among CRP from various animals.

**Reactivity with serum or CRP**	**Anti-CRP antibodies or antiserum**						
	**Human**	**Sheep**	**Rabbit**	**Dog**	**Cow**	**Goat**	**Horseshoe crab**
Human	**√**				**√**	**√**	**√**
Sheep		**√**				**√**	
Rabbit	**√**		**√**				**√**
Dog	**√**			**√**			
Cow	**√**				**√**	**√**	
Goat	**√**				**√**	**√**	
Rat							
Mouse		**√**					
Elephant				**√**			
Horse	**√**						
Monkey	**√**	**√**					
Cat	**√**						
Sheep	**√**				**√**		
Dogfish			**√**				
Snail							**√**
Horseshoe crab	**√**		**√**				**√**

*Only those animals are shown for which at least one immunological cross-reactivity is known. See the text for references*.

## CRP in an Arthropod

A protein that fits the definition of CRP is present in the hemolymph of the arthropod American horseshoe crab, *Limulus polyphemus* (5, 6, 10–13, 57–59). *Limulus* CRP binds to PCh in a Ca^2+^-dependent manner and precipitates PnC. *Limulus* CRP exhibits immunological cross-reactivity against snail CRP; the cross-reactivity against human CRP and rabbit CRP is weak. *Limulus* CRP is made of three types of subunits, each subunit having 218 amino acid residues, encoded by three homologous genes. The three subunits, which share an identical N-terminal sequence of 44 amino acid residues and a C-terminal sequence from amino acid residues 206–218, exist approximately in equimolar amount and are tightly associated. Hexagonal *Limulus* CRP, as revealed by electron microscopy, consists of two copies of each type of subunit. The positions of six half-cystines that form three intrachain disulfide bonds and the site of glycosylation are constant in all subunits. The molecular weight of *Limulus* CRP is ~300 kDa. The molecular weight of the subunits is ~25 kDa. Thus, there are 12 subunits in one *Limulus* CRP molecule, that is, two hexamers stacked together. The concentration of CRP in *Limulus* hemolymph is ~2.0 mg/ml. It remains to be determined whether *Limulus* CRP is an acute phase protein. *Limulus* CRP has also been shown to chelate the heavy metals mercury and cadmium, and hence playing a role in detoxification of heavy metals. Such a detoxification function of *Limulus* CRP is not known for human CRP. Uniquely, *Limulus* CRP has also been shown to exhibit Ca^2+^-independent binding to membranes mimicking the outer membrane of Gram-negative bacteria and then create pores in the lipid bilayer (5, 6, 10–13, 57–59).

## CRP in a Mollusc

Based on the reactivity with anti-*Limulus* CRP antibodies, CRP was detected in the hemolymph of the mollusc, giant African snail, *Achatina fulica* (14, 15, 60–62). *Achatina* CRP binds to PCh in a Ca^2+^-dependent manner. The molecular weight of *Achatina* CRP is ~400 kDa. *Achatina* CRP is glycosylated and has two types of subunits, of molecular weight 20 and 24 kDa. Although anti-*Limulus* CRP antibodies react with *Achatina* CRP, anti-*Achatina* CRP antibodies do not react with *Limulus* CRP. *Achatina* CRP is a constitutively expressed protein and one of the major components of the haemolymph with a normal level of ~2.0 mg/ml. Like *Limulus* CRP, *Achatina* CRP has also been shown to bind to heavy metals and it has been proposed that *Achatina* CRP may be utilized as a viable exogenous agent of cytoprotection against heavy metal-related toxicity. In addition, *Achatina* CRP has been found to be bacteriostatic against gram negative bacteria and bactericidal against gram positive bacteria (14, 15, 60–62).

## CRP in Fish

CRP was first found in the serum of a marine teleost fish, plaice, *Pleuronectes platessa* (16–19) and has been isolated and characterized mostly from teleost fish. Plaice CRP binds to PCh in a Ca^2+^-dependent manner and looks pentameric in its electron microscopic appearance. The molecular weight of plaice CRP is ~187 kDa, consisting of 10 non-covalently associated subunits arranged in two pentameric discs. There are two distinct subunits in plaice CRP; the difference in size between the subunits is due to carbohydrates since the heavier subunit is glycosylated while the lighter one is not. Plaice CRP is present at a concentration of 55 μg/ml and is not an acute phase protein because its serum concentration does not increase in response to turpentine. However, adrenal hormones and endotoxins do cause an increase in circulating CRP in plaice (16–19). A CRP-like protein has also been purified from the eggs of another marine teleost, lumpsucker, *Cyclopterus lumpus* (20, 21). The molecular weight of lumpsucker CRP is in the range of 125 and 150 kDa. Lumpsucker CRP consists of identical, non-covalently bound subunits of molecular weight 20–21.5 kDa.

CRP is also present in the sera of another teleost, the rainbow trout species, *Salmo gairdneri* and *Oncorhynchus mykiss* (22, 63–67). Trout CRP binds to PCh in a Ca^2+^-dependent manner, precipitates PnC, is a glycoprotein, and has 179 amino acid residues. According to one study, the molecular weight of rainbow trout CRP is 110 kDa, while that of the subunits is ~20 kDa. According to another study, the molecular weight of rainbow trout CRP is 81.4 kDa, it is a trimer, and composed of one monomer subunit (26.6 kDa) and one disulfide-linked dimer (43.7 kDa). However, there exists the possibility that a hexamer or a double-stacked hexamer was separated into two or four trimers. The CRP level in normal trout sera is in the range of 30–88 μg/ml. Trout CRP is an acute phase protein because its concentration increases in response to toxic chemicals and bacterial pathogens (22, 63–67). Cod, *Gadus morhua*, CRP that exhibits Ca^2+^-dependent binding to PCh, is glycosylated, and is a pentamer in its electron microscopic appearance. The size of the subunits in cod CRP varies in the range of 22–29 kDa (23, 24).

Among other teleosts, CRP has been isolated from the sera of eels *Anguilla anguilla* and *Anguilla japonica* (25, 26). Eel CRP binds to PCh in a Ca^2+^-dependent manner and agglutinates *S. pneumoniae*. The molecular weight of eel CRP is 120 kDa and the subunits are non-glycosylated, with a molecular weight of 24 kDa. The serum levels of eel CRP is ~1 μg/ml. CRP is present in channel catfish *Ictalurus punctatus* (27). Catfish CRP binds to PCh and precipitates PnC in a Ca^2+^-dependent manner. Catfish CRP is a non-glycosylated protein with a molecular weight of ~100 kDa. Electron microscopy has shown that catfish CRP has a planar pentagonal symmetry. The serum titer of catfish CRP follows an acute phase pattern in catfish injected with turpentine. Striped catfish, *Pangasianodon hypophthalmus*, CRP binds to PCh in a Ca^2+^-dependent manner, is a trimer of 28 kDa subunits, can exist as tetramers of trimers, is devoid of interchain disulfide bonds, is glycosylated, and agglutinates a few species of pathogenic bacteria (28). A CRP gene in ayu, *Plecoglossus altivelis*, has been identified (29). The expression of ayu CRP is upregulated following bacterial infection. Ayu CRP agglutinates both gram negative and positive bacteria in a Ca^2+^-dependent manner. Ayu CRP is not glycosylated, has a molecular weight of 25.2 kDa, and has 225 amino acid residues.

In cyprinids, the carp family fish rohu, *Labeo rohita*, CRP has been purified by their Ca^2+^-dependent binding to PCh (30, 68, 69). There are three types of glycosylated subunits in rohu CRP, and all three types of subunits move to identical position after desialylation and deglycosylation. Rohu CRP appears pentameric under electron microscope and is composed of identical subunits of molecular weight 33 kDa. Rohu CRP is an acute phase protein because its concentration in serum increases in response to heavy metal poisoning of water. In common carp *Cyprinus carpio*, CRP CRP displays Ca^2+^-dependent binding to phosphate monoesters (31, 37, 70–72). Common carp CRP is glycosylated, has 208 amino acid residues, and the molecular weight of the subunits is 27 kDa. A potential commercial use of CRP, which is constitutively expressed in common carp, is as a biomarker of health status in cultured carp. Serum level of CRP in common carp infected with some but not all pathogens increases several-fold, suggesting that common carp CRP is a minor acute phase protein. CRP has also been purified from the sera of major carp *Catla catla* (32, 33). Kinetic studies of metal intoxication in major carp indicated that a unique molecular variant of CRP is present in the serum at the peak level of acute phase induction, and this variant coexists with normal CRP. Major carp CRP is a glycoprotein, contains two non-identical subunits of molecular weight 22 and 29 kDa, and binds to PCh in a Ca^2+^-dependent manner. The electrophoretic mobility of the subunits is identical after desialylation and deglycosylation implying that the molecular variants vary in the glycan parts. The expression of CRP during the course of parasitic infection in the goldfish, *Carassius auratus*, was also determined (34); goldfish CRP, which has 214 amino acid residues and has subunits of 25.6 kDa, enhances complement-mediated killing of trypanosomes *in vitro*, and lysis increases after addition of immune serum.

Dogfish, *Mustelus canis*, CRP also binds to PCh in a Ca^2+^-dependent manner and precipitates PnC (35, 36, 73). Dogfish CRP has a molecular weight of ~250 kDa with subunits of molecular weight of ~25 kDa. Dogfish CRP probably exists as pentamers of two disulfide-linked dimers; however, the crystals of the protein were found to contain two hexamers in the asymmetric unit. Dogfish CRP is present at a concentration of 400 μg/ml. Dogfish CRP exhibits immunological cross-reactivity with rabbit CRP. The CRP gene from zebrafish, *Danio rerio*, a bony fish, has been cloned, expressed, protein purified, and crystallized (37, 38, 74, 75). There are seven CRP-like genes in zebrafish which are differentially expressed both normally and in acute phase and have anti-viral activities. Zebrafish CRP is trimeric, and each subunit has 208 amino acid residues. In tonguefish, a flat fish, CRP is composed of 210 amino acid residues with a subunit molecular weight of ~26 kDa (39). Expression of tonguefish CRP is upregulated by pathogen infection. Tonguefish CRP has been found to interact with both gram positive and negative bacteria.

In rockfish, *Sebastes taczanowskii*, CRP is a sex-limited protein (40). CRP is induced in the serum of males by estrogen administration. Serum levels in females during vitellogenic and gestation periods are about 1,000 times higher than those in the normal males. In the presence of Ca^2+^, rockfish CRP binds to PCh and agglutinates *S. pneumoniae*. In rockfish, two types of CRP are found, with molecular weights 160 and 152 kDa, with subunits of 30 and 26 kDa, respectively. Both subunits are glycosylated. In another species of rockfish, *Sebastes schlegelii*, CRP contains 208 amino acid residues with a molecular weight of 25 kDa (76). In Atlantic salmon *Salmo salar*, five CRP-like molecules are present (77). Salmon CRP has 208 amino acid residues, is not an acute phase protein, and only one of five CRP species is upregulated by cytokines.

## CRP in Birds, Reptiles, and Amphibians

So far, the presence of CRP has been investigated only in the fowl *Gallus gallus*. In chickens, CRP has 206 amino acid residues. The mRNA for CRP was found in many tissues from the fowl by using a probe derived from human CRP cDNA (78, 79). Although genes are present in lizards, the CRP protein has not been isolated and characterized from any reptile (77). Among amphibians, CRP has been isolated from the frog *Xenopus laevis* (41). Frog CRP has 210 amino acid residues. Xenopus CRP is present at an intermediate low level of ~1 μg/ml in the normal serum. Frog CRP level in the serum is not induced by turpentine. It is suggested that frog CRP represents a transitional period in the evolution of CRP, when host defenses switched from primitive innate immunity to the immune system. The constitutive functions of CRP gradually became less essential as a result of the development of a complex immune system (41).

## CRP in Mammals

CRP from cow, *Bos taurus*, has been purified from the serum (42, 43, 80). Unlike human CRP, bovine CRP does not precipitate PnC. However, bovine CRP exhibits immunological cross-reactivity with human, goat and sheep CRP. Bovine CRP is a pentameric molecule with a molecular weight of ~100 kDa and is composed of five identical non-glycosylated subunits of molecular weight of ~23 kDa. Each subunit has one intrachain disulfide bond. The pentameric structure of bovine CRP was seen by electron microscopy. The concentration of bovine CRP is in the range of 5–40 μg/ml and it is not an acute phase protein (42, 43, 80).

CRP from dogs, *Canis lupus*, exhibits Ca^2+^-dependent binding to PCh (44, 45, 81–88). Dog CRP has the typical cyclic pentameric disc-like structure in its electron microscopic appearance, although the pentamer stacks. Dog CRP has 204 amino acid residues and a molecular weight of ~100 kDa. Dog CRP is composed of five subunits of ~20 kDa with an intrachain disulfide bond in each subunit. In another study, the molecular weight of dog CRP was estimated to be ~156 kDa. In one study, two isotypes of CRP with different molecular weights, 22 and 25 kDa were found, with the 25 kDa subunit glycosylated. Two of the five subunits in the pentamer were glycosylated. Antibodies to human CRP react with dog CRP, but antibodies to dog CRP do not react with human CRP. However, antibodies to dog CRP was used to detect CRP in elephants. CRP Normal healthy dogs contain ~5–60 μg/ml of CRP but, following a stimulus, CRP behaves as an acute phase protein (44, 45, 81–88).

Guinea pig CRP has 206 amino acid residues and has not been characterized fully, but the gene has organization typical of the CRP genes of other mammals. Guinea pig CRP is not an acute phase protein (89). Harbor seal, *Phoca vitulina*, CRP binds to PCh in a Ca^2+^-dependent manner and has a molecular weight of ~25 kDa (46). A CRP-like protein has also been isolated from goat serum (47). Direct binding of goat CRP to PCh has not been shown; however, fluid phase PCh inhibits the Ca^2+^-dependent binding of goat CRP to its ligand agarose. Goat CRP is composed of five identical, glycosylated, non-covalently associated subunits, each of molecular weight ~24 kDa. Goat CRP possesses immunological cross-reactivity with human, cow and sheep CRP. Like in cows and guinea pigs, CRP in goats is not an acute phase protein (47). CRP in horses has pentameric structure as revealed by electron microscopy and binds to PCh in a Ca^2+^-dependent manner (48, 90). Horse CRP has 204 amino acid residues and a molecular weight of ~118 kDa. Horse CRP is composed of five identical, non-glycosylated and non-covalently associated subunits with molecular weight of ~23 kDa. Equine CRP displays immunochemical cross-reactivity with human CRP. In horses, CRP is an acute phase protein (48, 90). Monkey CRP has 206 amino acid residues and precipitates PnC; however, monkey CRP was first isolated by Ca^2+^-dependent binding to organic monophosphates. Monkey CRP reacts with anti-sheep CRP antibodies but not with anti-dog CRP antibodies (91). CRP from pigs has 203 amino acid residues but not been characterized fully yet. Porcine CRP is an acute phase protein (92, 93).

A CRP-like protein named female protein (FP) was found in Syrian and Armenian hamsters (49–51, 94–96). FP is a prominent serum constituent of normal female hamsters but is under hormonal control in males. However, in normal adult male hamsters, FP in serum increases only about 5-fold during an acute phase response, in contrast to human CRP which may increase 1,000-fold or more. FP has a pentameric structure as indicated by electron microscopy and binds to PCh in a Ca^2+^-dependent manner. FP has 206 amino acid residues with a molecular weight in the range of 128–150 kDa. The molecular weight of each of the five non-covalantly assembled glycosylated subunits is in the range of 26–30 kDa; each subunit contains a single intrachain disulphide bond. In the presence of Ca^2+^, FP aggregates, probably to form decamers (49–51, 94–96). In mice (97–100), CRP is not an acute phase protein. Murine CRP agglutinates several strains of gram-positive bacteria *in vitro*. Murine CRP has 206 amino acid residues. Protein modeling has demonstrated that adaptively selected amino acid residues in murine CRP lie in the ligand-binding region and contact region between subunits (97–100).

Rabbit CRP reacts with PCh and precipitates PnC in a Ca^2+^-dependent manner (52, 53, 91, 101–120). CRP was found localized at sites of inflammation in rabbits and was not observed at the inflammatory site before appearance in the blood. The concentration of rabbit CRP in the serum is ~1.5 μg/ml. Investigation of rabbit CRP provided evidence that CRP molecules expressing a structure and antigenicity that are distinct from native CRP occurs *in vivo*, and that such molecules accumulate at sites of inflammation. Rabbit CRP has 205 amino acid residues and is pentameric. The molecular weight of rabbit CRP lies between 115–140 kDa. The subunit size is 23.5 kDa. For precipitation of the PCh-ligands, only the binding of the phosphoryl group of PCh to rabbit CRP is required, unlike for human CRP where binding to both the phosphoryl and cationic groups of PCh are needed for precipitation. Transgenic mice expressing rabbit CRP are partially protected from a lethal challenge of endotoxin or platelet activating factor. Rabbit CRP is capable of activating complement when bound to a ligand; however, complement activation is not required to mediate protection against either endotoxins or platelet activating factor. Immobilized rabbit CRP binds to low-density lipoprotein (LDL) also. In rabbits, CRP has been found associated with the progression of atherosclerosis (52, 53, 101–120).

Rat CRP is unique when compared to CRP from other mammalian species (54, 111, 112, 121–133). Rat CRP has five subunits arranged as a cyclic pentamer and is the only mammalian CRP which is glycosylated and contains a covalently linked dimer in its pentameric structure. Rat CRP binds to PCh in a Ca^2+^-dependent manner but does not precipitate PnC. The non-glycosylated rat CRP is also able to bind to PCh. Rat CRP is made up of 206 amino acid residues. One pair of the subunits per molecule is linked by two interchain disulphide bonds, that is, the five subunits are not non-covalently associated. The other three subunits have intrachain disulfide bonds. Rat CRP is present at a concentration of 0.3–0.5 mg/ml and is not an acute phase protein. Immobilized rat CRP is capable of binding to LDL also in a Ca^2+^-dependent and PCh-inhibitable manner; however, the binding ability of rat CRP to PCh is not a sufficient requirement for the interaction between rat CRP and LDL. A sialic acid moiety must also be present on rat CRP for binding to LDL. LDL is modified once it is complexed with rat CRP. Like Limulus CRP, rat CRP has also been shown to play a role in detoxification of heavy metals such as mercury (54, 111, 112, 121–133).

Mink presents an unusual case (134): it is called mink SAP. However, mink SAP is not glycosylated unlike SAP in other mammals. Mink SAP binds to PCh also, unlike SAP in other mammals. The molecular weight of mink SAP subunits is ~26 kDa. The presence of mRNA for CRP has also been found by using a probe derived from human CRP cDNA in many tissues from Asian elephant *Elephas maximus* (135, 136) and ground squirrel *Spermophilus richarsonii* (137). CRP is also present in elephant seal *Mirounga angustirostris* (138). A gene sequence for CRP is also found in marsupial genome, red-tailed phascogale *Phascogale calura* (139).

## Human CRP

In humans, CRP is a major acute phase protein whose concentration may increase more than 1,000-fold in severe inflammatory states (9). Human CRP is a pentameric protein composed of five identical non-covalently bound subunits of 206 amino acid residues with a molecular weight of ~23 kDa. Human CRP binds to PCh in a Ca^2+^-dependent manner. There are five PCh-binding sites, one located on each subunit. Each subunit binds two Ca^2+^ ions (55, 140). In human CRP, Glu^81^ in the PCh-binding site interacts with the nitrogen atom of choline in PCh, Phe^66^ interacts with three methyl groups of choline, and Thr^76^ is critical for creating the appropriately sized pocket to accommodate PCh. The phosphate group of PCh directly coordinates with the two Ca^2+^ bound to CRP. Using synthetic peptides derived from CRP, direct binding of Ca^2+^ to a peptide of residues 134–148 has been shown. Crystallography of CRP has demonstrated that two Ca^2+^ ions are co-ordinated by Asp^60^, Asn^61^, and by residues Glu^138^, Gln^139^, Asp^140^, Glu^147^, and Gln^150^ in a loop (55, 141–144). Once CRP is bound to a PCh-containing ligand, it activates the classical complement pathway. Residues Asp^112^ and Tyr^175^ play critical roles in the formation of the C1q-binding site in CRP (145–147).

Acidic pH transforms native pentameric CRP into another pentameric configuration, called as non-native CRP, which exposes a hidden ligand-binding site for non-PCh ligands, and which enables CRP to bind to immobilized, denatured and aggregated proteins. For example, CRP does not bind to oxidized LDL (ox-LDL) at physiological pH but gains the ability to bind to ox-LDL at acidic pH (148). H_2_O_2_-treated CRP also gains a ligand recognition property not exhibited by native CRP, indicating that H_2_O_2_, like acidic pH, is another modifier of the structure and ligand recognition function of CRP (149). Immobilization of CRP or mutagenesis of Glu^42^ in the inter-subunit contact region in pentameric CRP also convert CRP into molecules that bind to a variety of immobilized, denatured and aggregated proteins (148–151). A possible binding site in non-native CRP for immobilized, denatured and aggregated proteins could be formed involving the single intrinsically disordered region present in CRP (152). It has been shown that when CRP dissociates into its monomers, monomeric CRP recognizes such protein ligands through the intrinsically disordered region (152).

CRP is a multifunctional component of the human innate host defense machinery. In mouse models of pneumococcal infection, transgenic or passively administered human CRP has been shown to be protective against lethal infection with *S. pneumoniae* (153–160). Similarly, CRP may be an atheroprotective molecule, as shown by using transgenic CRP in animal models of human like atherosclerosis (161–166). CRP has been found deposited at sites of inflammation, indicating the presence of non-native CRP *in vivo*. The functions of CRP at sites of inflammation have not been defined yet; however, it has been suggested that a structural change in CRP and the resulting shift from the ligand recognition function of CRP in its native conformation to another ligand recognition function in its non-native conformation occurs at sites of inflammation (151, 152, 167).

Interestingly, human CRP also possesses sites for glycosylation, although the sites are hidden in native CRP (168–170). When CRP was isolated from patients with six different pathological conditions, CRP was found to be differentially glycosylated. A few amino acids at the N-terminus and a few amino acids near the C-terminus are missing in glycosylated human CRP. The cleavage of these peptides from CRP exposes two potential sites of glycosylation and these sites are located on CRP on the face opposite to the PCh-binding face of CRP. It has been proposed that glycosylated CRP has a protective role toward the clearance of damaged erythrocytes in diseases.

## Evolution of CRP

As the nature of CRP gene expression evolved from a constitutively expressed protein in arthropods to an acute phase protein in humans, the definition of CRP also became distinctive. In humans, CRP can easily be distinguished from other homologous proteins such as SAP, but this is not the case in invertebrates. For invertebrates, it has always been difficult to define a protein as either CRP or SAP because of the similarities in their structures and functions. Also, whereas a single CRP gene is present in the human, multiple genes are present in some species, such as *Limulus*.

There is sequence similarity and homology among the known functional sites of CRP from all species (Figure 1). The PCh-binding property of CRP has been conserved. However, employing animal models of pneumococcal infection it has been shown that the PCh-binding property of human CRP is not the only relevant ligand recognition function of human CRP. Apparently, another ligand-binding property of CRP, such as recognition of complement regulator protein factor H by CRP in its non-native conformation, is responsible for its host defense functions (160). Because some ancient CRP molecules including bovine CRP do not bind PCh, it has been proposed that the recognition of PCh by CRP is less relevant to the role of the protein than its interaction with other ligands (42, 151, 160).

Human CRP in its non-native structural conformation expresses the capability to bind to deposited and conformationally altered proteins and which can be achieved by several means including treatment of CRP with acidic pH (151). The ligand-binding property of human CRP in its non-native structure has implications for toxic and inflammatory conditions and favors the conservation of CRP throughout evolution. It seems that the host-defense functions of CRP evolved to expose a ligand-binding site only when needed, that is, an inflammatory microenvironment would have to be sensed by CRP first and that CRP would change its structure to execute a function. It is not known, however, whether CRP from invertebrates also exhibits structure-based ligand-binding properties. A recent study has shown that *Limulus* CRP is capable of binding to immobilized ox-LDL without being pre-treated with acidic pH (171). During evolution, changes in the intrachain disulfide and interchain disulfide bonds and the changes in the glycosylation status of CRP may also be responsible for the structure-function relationships of CRP in various species.

## Conclusions

CRP evolved as a component of and along with the development of the entire immune system. Both structure and function of CRP have evolved; however, more studies on CRP from all invertebrates and vertebrates are needed to understand fully the reasons behind the evolution of CRP. Structure-function relationships of CRP from most animals are unknown. We know that the ligand-binding properties of *Limulus* CRP are not identical to that of native human CRP but overlap the ligand-binding properties of non-native pentameric human CRP that can be generated at inflammatory microenvironments (143, 158). Since the ligand recognition functions of CRP lead to effector functions, it is important to understand the biology of ancient CRP molecules because the knowledge could be useful for immunodeficient individuals: all humans have CRP, but it is not known whether human CRP is functional in all humans.

## Author Contributions

All authors listed have made a substantial, direct and intellectual contribution to the work, and approved it for publication.

### Conflict of Interest Statement

The authors declare that the research was conducted in the absence of any commercial or financial relationships that could be construed as a potential conflict of interest.

## Abbreviations

CRP: C-reactive protein
FP: female protein
LDL: low density lipoprotein
PCh: phosphocholine
PnC: pneumococcal C-polysaccharide
SAP: serum amyloid P component.
